# The representative COVID-19 cohort Munich (KoCo19): from the beginning of the pandemic to the Delta virus variant

**DOI:** 10.1186/s12879-023-08435-1

**Published:** 2023-07-13

**Authors:** Ronan Le Gleut, Michael Plank, Peter Pütz, Katja Radon, Abhishek Bakuli, Raquel Rubio-Acero, Ivana Paunovic, Friedrich Rieß, Simon Winter, Christina Reinkemeyer, Yannik Schälte, Laura Olbrich, Marlene Hannes, Inge Kroidl, Ivan Noreña, Christian Janke, Andreas Wieser, Michael Hoelscher, Christiane Fuchs, Noemi Castelletti, the KoCo19/ORCHESTRA-study group, Mohamed Ibraheem Mohamed Ahmed, Emad Alamoudi, Jared Anderson, Valeria Baldassarre, Maximilian Baumann, Marc Becker, Franziska Bednarski, Marieke Behlen, Olimbek Bemirayev, Jessica Beyerl, Patrick Bitzer, Rebecca Böhnlein, Isabel Brand, Anna Brauer, Vera Britz, Jan Bruger, Franziska Bünz, Friedrich Caroli, Josephine Coleman, Lorenzo Contento, Alina Czwienzek, Flora Deák, Maximilian N. Diefenbach, Paulina Diepers, Anna Do, Gerhard Dobler, Jürgen Durner, Tabea Eser, Ute Eberle, Judith Eckstein, Philine Falk, Manuela Feyereisen, Volker Fingerle, Stefanie Fischer, Jonathan Frese, Felix Forster, Günter Fröschl, Otto Geisenberger, Mercè Garí, Marius Gasser, Sonja Gauder, Raffaela Geier, Kristina Gillig, Christof Geldmacher, Keisha Gezgin, Leonard Gilberg, Kristina Gillig, Philipp Girl, Elias Golschan, Vitus Grauvogl, Jessica Michelle Guggenbuehl Noller, Elena Maria Guglielmini, Pablo Gutierrez, Anselm Haderer, Celina Halfmann, Lena Hartinger, Timm Haselwarter, Jan Hasenauer, Alejandra Hernandez, Luca Heller, Arlett Heiber, Matthias Herrmann, Leah Hillari, Stefan Hillmann, Christian Hinske, Janna Hoefflin, Tim Hofberger, Michael Höfinger, Larissa Hofmann, Sacha Horn, Kristina Huber, Christian Janke, Lilian Karger, Ursula Kappl, Antonia Keßler, Zohaib Khan, Charlotte Kiani, Isabel Klugherz, Norah Kreider, Johanna Kresin, Arne Kroidl, Pratik Kunder, Magdalena Lang, Clemens Lang, Silvan Lange, Ekaterina Lapteva, Michael Laxy, Reiner Leidl, Leopold Liedl, Felix Lindner, Xhovana Lucaj, Elisabeth Lucke, Fabian Luppa, Alexandra Sophie Nafziger, Alexander Maczka, Petra Mang, Alisa Markgraf, Paula Matcau, Rebecca Mayrhofer, Anna-Maria Mekota, Dafni Metaxa, Emily Mohr, Hannah Müller, Katharina Müller, Nathalia Nascimento, Kasimir Niermeyer, Sophia Nikolaides, Leonie Pattard, Claire Pleimelding, Michel Pletschette, Viona Poll, Stephan Prückner, Kerstin Puchinger, Konstantin Pusl, Elba Raimúndez, Julius Raschka, Jakob Reich, Christina Reinkemeyer, Camilla Rothe, Viktoria Ruci, Elmar Saathoff, Nicole Schäfer, Paul Schandelmaier, Benedikt Schluse, Annika Schneider, Lara Schneider, Sophie Schultz, Mirjam Schunk, Lars Schwettmann, Josefin Sedlmeier, Linda Sintu-Sempta, Alba Soler, Peter Sothmann, Katharina Strobl, Aida Strüber, Laura Strüber, Jeni Tang, Fabian Theis, Verena Thiel, Eva Thumser, Niklas Thur, Julian Ullrich, Vincent Vollmayr, Emilia Von Lovenberg, Jonathan Von Lovenberg, Carsten Vos, Julia Waibel, Claudia Wallrauch, Nikolas Weigl, Roman Wölfl, Julia Wolff, Pia Wullinger, Tobias Würfel, Patrick Wustrow, Sabine Zange, Eleftheria Zeggini, Anna Zielke, Thorbjörn Zimmer, Thomas Zimmermann, Anna Zielke, Lea Zuche

**Affiliations:** 1 Institute of Computational Biology, Helmholtz Munich, German Research Centre for Environmental Health, 85764 Neuherberg, Germany; 2 Core Facility Statistical Consulting, Helmholtz Munich, German Research Centre for Environmental Health, 85764 Neuherberg, Germany; 3 Division of Infectious Diseases and Tropical Medicine, University Hospital, LMU Munich, 80802 Munich, Germany; 4 Robert Koch Institute, Nordufer 20, 13353 Berlin, Germany; 5 Institute and Outpatient Clinic for Occupational, Social and Environmental Medicine, University Hospital, LMU Munich, 80336 Munich, Germany; 6 Centre for International Health (CIH), University Hospital, LMU Munich, 80336 Munich, Germany; 7 Comprehensive Pneumology Centre (CPC) Munich, German Centre for Lung Research (DZL), 89337 Munich, Germany; 8 Centre for Mathematics, Technische Universität München, 85748 Garching, Germany; 9 Life and Medical Sciences Institute, University of Bonn, 53115 Bonn, Germany; 10 German Centre for Infection Research (DZIF), Partner Site, Munich, Germany; 11 Fraunhofer Institute for Translational Medicine and Pharmacology ITMP, Immunology, Infection and Pandemic Research, 80799 Munich, Germany; 12 Max Von Pettenkofer Institute, Faculty of Medicine, LMU Munich, 80336 Munich, Germany; 13 Faculty of Business Administration and Economics, Bielefeld University, 33615 Bielefeld, Germany; 14 Institute of Radiation Medicine, Helmholtz Munich, German Research Centre for Environmental Health, 85764 Neuherberg, Germany

**Keywords:** COVID-19, SARS-CoV-2, Population-based cohort study, Sero-prevalence, Sero-incidence, Vaccination status, Breakthrough infections, ORCHESTRA

## Abstract

**Background:**

Population-based serological studies allow to estimate prevalence of SARS-CoV-2 infections despite a substantial number of mild or asymptomatic disease courses. This became even more relevant for decision making after vaccination started. The KoCo19 cohort tracks the pandemic progress in the Munich general population for over two years, setting it apart in Europe.

**Methods:**

Recruitment occurred during the initial pandemic wave, including 5313 participants above 13 years from private households in Munich. Four follow-ups were held at crucial times of the pandemic, with response rates of at least 70%. Participants filled questionnaires on socio-demographics and potential risk factors of infection. From Follow-up 2, information on SARS-CoV-2 vaccination was added. SARS-CoV-2 antibody status was measured using the Roche Elecsys® Anti-SARS-CoV-2 anti-N assay (indicating previous infection) and the Roche Elecsys® Anti-SARS-CoV-2 anti-S assay (indicating previous infection and/or vaccination). This allowed us to distinguish between sources of acquired antibodies.

**Results:**

The SARS-CoV-2 estimated cumulative sero-prevalence increased from 1.6% (1.1-2.1%) in May 2020 to 14.5% (12.7-16.2%) in November 2021. Underreporting with respect to official numbers fluctuated with testing policies and capacities, becoming a factor of more than two during the second half of 2021. Simultaneously, the vaccination campaign against the SARS-CoV-2 virus increased the percentage of the Munich population having antibodies, with 86.8% (85.5-87.9%) having developed anti-S and/or anti-N in November 2021. Incidence rates for infections after (BTI) and without previous vaccination (INS) differed (ratio INS/BTI of 2.1, 0.7-3.6). However, the prevalence of infections was higher in the non-vaccinated population than in the vaccinated one. Considering the whole follow-up time, being born outside Germany, working in a high-risk job and living area per inhabitant were identified as risk factors for infection, while other socio-demographic and health-related variables were not. Although we obtained significant within-household clustering of SARS-CoV-2 cases, no further geospatial clustering was found.

**Conclusions:**

Vaccination increased the coverage of the Munich population presenting SARS-CoV-2 antibodies, but breakthrough infections contribute to community spread. As underreporting stays relevant over time, infections can go undetected, so non-pharmaceutical measures are crucial, particularly for highly contagious strains like Omicron.

**Supplementary Information:**

The online version contains supplementary material available at 10.1186/s12879-023-08435-1.

## Background

**Fig. 1:**
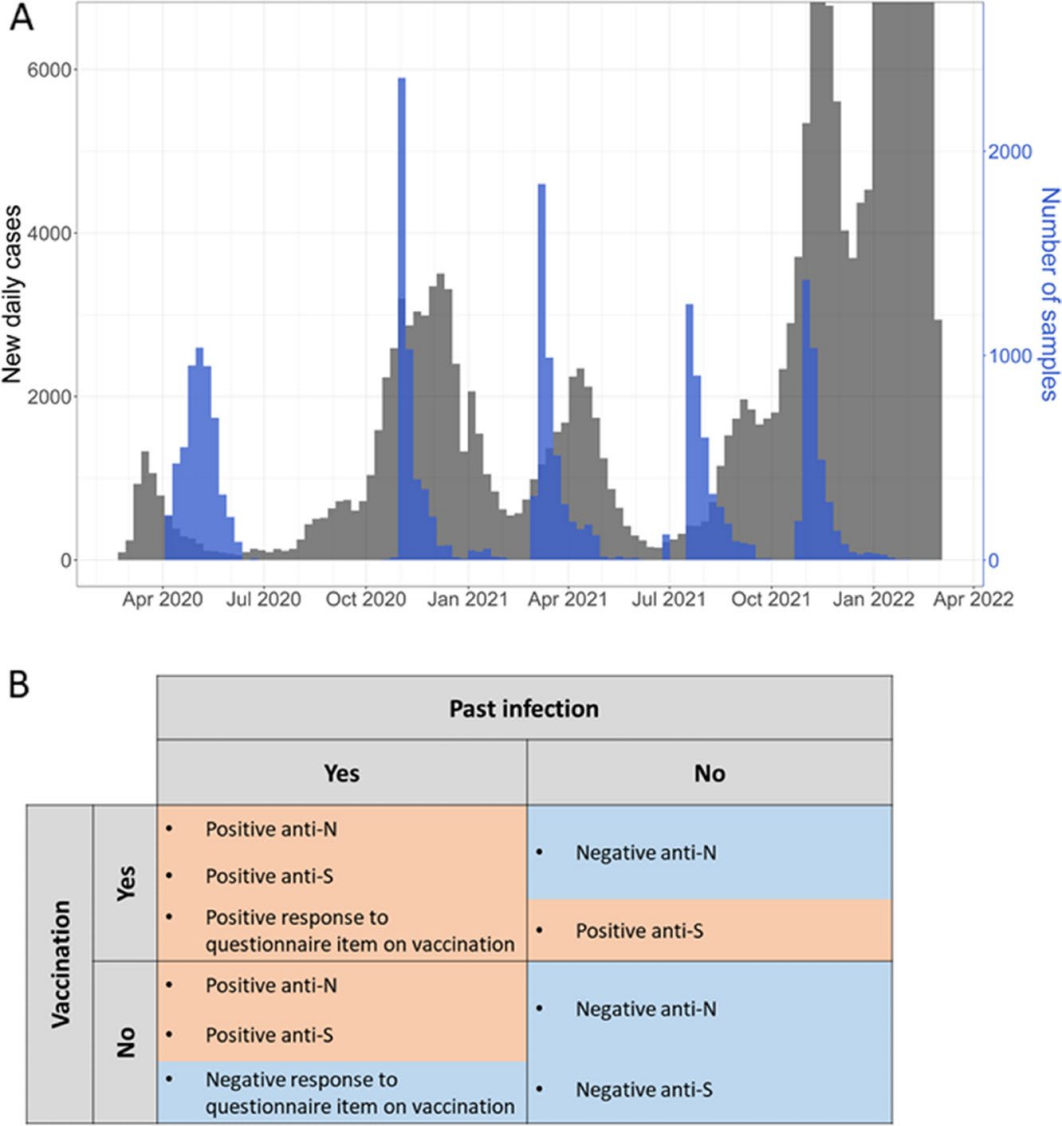
Epidemic evolution in Munich with description of the sample analysis. **A** Black: number of new daily SARS-CoV-2 cases officially reported by the Robert Koch Institute (RKI). Blue: number of blood/DBS samples of the KoCo19 collected daily. **B** Description of the lab analysis. With anti-N, anti-S and the response to the questionnaire item on vaccination it was possible to define the participants as: infected and vaccinated, infected and non-vaccinated, non-infected and vaccinated and non-infected and non-vaccinated. Blue shaded regions denote a negative response while orange regions a positive one

SARS-CoV-2 became pandemic mid-March 2020, within three months after the first report on 31st of December, 2019 in the city of Wuhan, Hubei province, China [1, 2]. In Germany, the first COVID-19 cases were observed in the municipality of Munich in late January 2020 [3]. Since then, the number of infections has been one of the predominant topics for political and social life [4, 5]. Looking at the pandemic in Munich in the time-frame between February 2020 and April 2022, four waves of infection can be identified (Fig. 1A):

- First wave: late January – mid June 2020
- Second wave: mid June 2020 – mid February 2021;
- Third wave: mid February 2021 – end July 2021;
- Fourth wave: end of July 2021 – after the end of the analysed period.

In the first wave, the main non-pharmaceutical interventions applied were to reduce contacts in the whole city of Munich followed by a lifting of the restrictions with still severe contact reductions. During this early phase of the pandemic, PCR tests were scarce good, and we suspect that only few chance finds entered the official statistics. In the second wave, contacts between people were reduced from June to October 2020, followed by stronger regulations, including FFP2 mask obligation. At the end of December 2020, only twelve months after the start of the pandemic, effective vaccines were introduced in Germany [6], preventing infection or at least reducing symptoms [7]. In parallel, the test capacity increased: starting in July 2020, the Bavarian state (including Munich) provided access to free PCR tests for all citizens, even without symptoms without a limit per person [8]. Antigen rapid tests became available nationwide for institutions like nursing homes or schools towards the end of 2020. By contact tracing more asymptomatic infected individuals could be identified [9–11]. In the third wave, the lock-down from the previous wave still continued with the so-called "emergency brake" starting in mid-April 2021: stronger contact reduction, night-time curfew and closure of many stores [12]. During this wave, the first new virus variant of SARS-CoV-2 was observed [13]: in early March 2021, the Alpha variant (B.1.1.7 variant) was detected in more than 40% of tested positive cases in Germany [14]. From early 2021 on, the testing capacity was further increased nationwide, and antigen test became available for home use [15, 16].Such low-threshold access to testing supposedly facilitated detecting asymptomatic cases, which entered the official numbers after PCR confirmation. The fourth wave of the pandemic started in Munich with almost all cases classified as Delta (B.1.617.2) variant. Further relaxations were possible in the summer breaks from July 2021: more visitors at outdoor and cultural events, restaurants could stay open longer, mask rules were relaxed, bars could reopen [17, 18]. In October 2021, even clubs were allowed to open again [19].

Decisions on non-pharmaceutical interventions were mostly taken under the guidance of official case reports, which were shown to underestimate the true case numbers especially at the beginning of the pandemic, when testing capacity was still low [20]. In order to gain a better understanding of the true case numbers, we started the prospective Munich COVID-19 cohort (KoCo19) in April 2020 including 5313 participants living in private households. In this population-based cohort study we measured SARS-CoV-2 antibody prevalence at the following times of the pandemic (Fig. 1A):

- May 2020 at the peak of the first wave in Germany,
- December 2020, at the beginning of the second wave,
- March 2021, at the peak of the third wave and at the beginning of the vaccination campaign for the general population,
- August 2021, at the end of the third wave with around 68% of the general population 14 years or older being vaccinated against SARS-CoV-2,
- November 2021, in the middle of the fourth wave and before the spread of the Omicron variant started in Germany.

To the best of our knowledge, KoCo19 is the SARS-CoV-2 cohort with the longest follow-up time in the world. On December 1st, 2020, the KoCo19 cohort joined the ORCHESTRA (Connecting European Cohorts to Increase Common and Effective Response to SARS-CoV-2 Pandemic) project. During the whole pandemic, KoCo19 results were used to advise political decision making.

We here present the evolution of SARS-CoV-2 cumulative sero-positivity in the Munich general population 14 years and older over time. Furthermore, we report on risk factors for SARS-CoV-2 infection over time. The data described here were not published elsewhere.

## Methods

### Study population and field work

#### Baseline and follow-up questionnaires

A detailed description of the baseline study can be found in [20, 21]: We randomly sampled the Munich cohort of private households between April 5th and June 12th, 2020. Only household members 14 years and older who gave written informed consent were included in the cohort. For participants younger than 18 years, informed consent was obtained from the parents as well as the participants themselves.

Analyses use information from baseline individual and household questionnaires and from individual follow-up questionnaires. The different questionnaires were already described in detail [20], and included information on: socio-demographics, country of birth, smoking status, chronic conditions, general health, household size, living area per inhabitant, household type, housing type, self-estimated health-related risk taking behaviour, personal contacts, number and intensity of leisure time activities before the pandemic (in February 2020), number and intensity of leisure time activities two weeks prior to the follow-up questionnaire. Starting from Follow-up 2, we also asked about SARS-CoV-2 vaccination including the number of vaccinations, type of vaccine and date of vaccination.

#### Baseline and follow-ups SARS‑CoV‑2 antibody study

At recruitment, a serum sample was gathered for 5313 household members 14 years and older. Thereafter, four antibody follow-ups were conducted in December 2020 [20], March 2021, August 2021 and November 2021 (Fig. 1A). Follow-ups were performed by sending out boxes with a self-sampling kit to take a capillary blood sample (dry blood spot; DBS). A detailed description of the DBS analysis procedure can be found in [22]. When self-DBS collection was impossible, participants were invited to give serum and DBS at our study centre.

For the measurements at baseline [23] and Follow-up 1, only the Elecsys® Anti-SARS-CoV-2 anti-N (Roche) (hereafter called Ro-N-Ig) assay was used for antibody detection after infection. From Follow-up 2 on, in addition, also the Elecsys® Anti-SARS-CoV-2 anti-S (Roche) (hereafter called Ro-RBD-Ig) assay was applied. This was necessary to distinguish antibodies due to infection (i.e., anti-S and anti-N present) and antibodies only due to vaccination (i.e., only anti-S present) (Fig. 1B).

For the measurement with full blood sampling, an optimised cut-off of 0.4218 for Ro-N-Ig was applied to indicate sero-positivity [23]. Estimates of sensitivity and specificity of blood Ro-N-Ig compared to reverse-transcription polymerase chain reaction (RT-PCR) were used to adjust the prevalence.

Taking full blood samples as ground truth, sensitivity and specificity of the DBS anti-N method were 99.2% and 98.7%, respectively, applying a cut-off of 0.105 [22]. Based on our internal validation cohort (data not shown here), only samples with Ro-RBD-Ig larger than or equal to 0.115 were considered positive (regarding anti-S) for vaccination and/or infection. Similarly, the DBS anti-S method had sensitivity and specificity of 96.6% and 97.8%, respectively. Since sensitivity and specificity of both tests turned out high, no additional adjustment for sensitivity and specificity was applied. The cut-offs for blood samples, as well as DBS samples, along with their sensitivity and specificity, were determined based on cohorts randomly selected using serology rather than symptom severity. This approach ensured that the assays are suitable for detecting milder community infections [22, 23].

Using the serological values in combination with questionnaire information, we were able to classify participants into the following groups (Fig. 1B):

- Non-vaccinated, non-infected: negative in both anti-S and anti-N antibodies;
- Vaccinated, non-infected: positive in anti-S and negative in anti-N antibodies;
- Non-vaccinated, infected: positive in both anti-S and anti-N antibodies, negative response to the questionnaire item on vaccination;
- Vaccinated and infected: positive in both anti-S and anti-N antibodies, positive response to questionnaire item on vaccination.

### Statistics

All statistical analyses were performed using the softwares R (version 4.1.3, R Development Core Team, 2021) and Python (version ≥ 3.8.5).

**Fig. 2:**
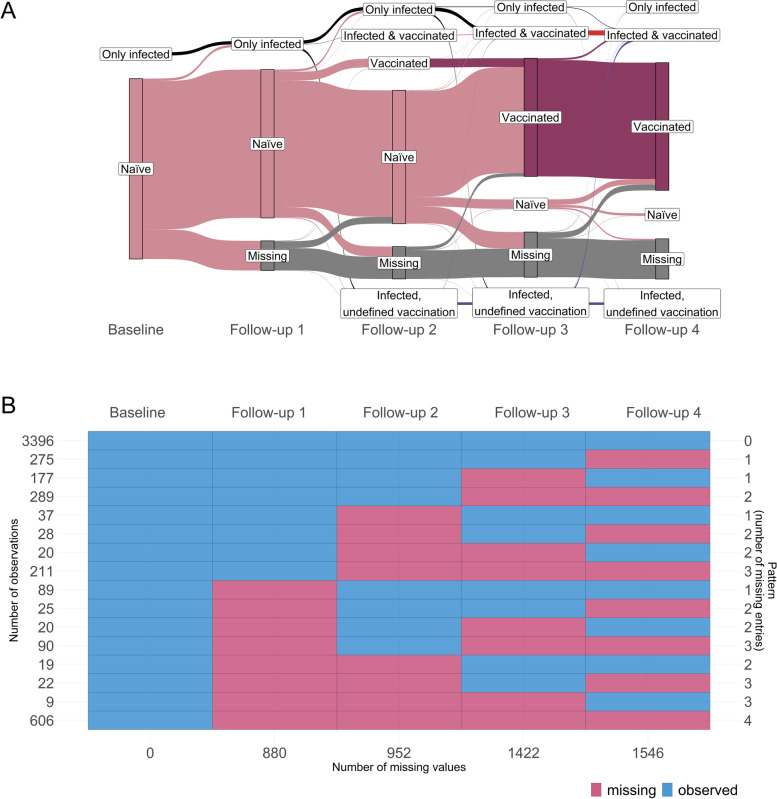
Cohort description based on the ever-positive principle, i.e. anti-N sero-positivity remains for all rounds after sero-conversion, independently of other blood results or if missing. **A** Change of serological status of participants: only infected (anti-N ever positive and stated to be non-vaccinated in the questionnaire), naïve (anti-N and anti-S always negative), vaccinated (only anti-S ever positive), infected & vaccinated (in previous round only anti-S positive, or stated to be vaccinated in the questionnaire), infected without information on vaccination (infected, undefined vaccination) and non-responders/missing. **B** Observed responder behaviours. Left legend: number of participants. Right legend: number of missing rounds. Bottom legend: number of missing samples per round

After observed sero-conversion, antibody levels were imputed positive in all follow-ups, independently of the actual results of the round or in case of missingness („ever positiveness “, Fig. 2A). We thus disregard potential anit-N waning. Our definition allows us to estimate the cumulative sero-prevalence in the considered population, which in turn we take as a proxy for cumulative infections and compare to the official number of positive cases reported by the authorities, neglecting reinfections. For simplicity, we in the following suppress the word “cumulative” as a specification of the estimated sero-prevalence. In order to estimate the population prevalence, sero-prevalence estimates (adjusted and unadjusted for the sensitivity and specificity of the test) were computed using a weighting scheme. First, sampling weights for each participant at baseline were calculated according to the sampling design of the cohort [21]. These weights were then corrected for the attrition observed at each follow-up, modelling the underlying non-response mechanism [24]. The resulting weights were finally calibrated on the updated Munich structure at each round regarding age, sex, country of birth, presence of children in the household and single member households distributions [25]. For the last three follow-ups (March, August and November 2021), information on the vaccination status of the participants was assessed via questionnaires. The missing values (30% for Follow-up 2, 27% for Follow-up 3 and 8% for Follow-up 4) were imputed via multiple imputation (*m* = 100) crossing for each round the vaccination status with the information on the immune response (Ro-N-Ig and Ro-RBD-Ig results). The probability $p$ of being vaccinated was estimated for each of the four anti-N and anti-S combinations for each of the imputed datasets and each Follow-up 2 to 4, see e.g. the values of one imputed dataset for Follow-up 4 in Table 1. The results for Follow-up 3 are comparable to these ones. At the beginning of the vaccination campaign (Follow-up 2), the probabilities to be vaccinated were lower, especially for anti-S and anti-N positive ($p=0.06$) with mostly only infected (and non vaccinated) persons.

**Table 1:** Estimated probabilities to be vaccinated used for the imputation of the vaccination status during Follow-up 4

		Anti-N	
		Positive	Negative
Anti-S	Positive	$p=0.94$	$p=0.99$
	Negative	$p=0$	$p=0.19$

Anti-S negative may occur after vaccination in case of a delayed or an absence of antibody response. Moreover, Ro-RBD-Ig (anti-S) with a cut-off at 0.115 does not provide 100% sensitivity and specificity

The imputation was performed using a Bernoulli distribution with probability $p$ for each participant with missing information.

Considering both Ro-RBD-Ig results and the questionnaire data, in the last two follow-ups 93% and 97%, respectively, of the participants could be assumed vaccinated. In contrast, the city of Munich reported that approximately 68% and 76%, respectively, of the population older than 14 years have been vaccinated [26]. The calibration of the cohort results is hence of crucial importance. The variance associated with the calibrated sero-prevalence estimates was computed using linearisation [25] and residual [25, 27] techniques. This variance accounts for the uncertainty due to the different stages of the sampling design (selection of the constituencies and of the households), the non-response mechanism [28] and the calibration process. As a sensitivity analysis, unweighted sero-prevalence estimates were also computed together with their uncertainty. The variance was determined by a nonparametric cluster bootstrap procedure that accounts for household clustering [29]. The sero-prevalence estimates were calculated in each of the 5000 bootstrap samples (sampling of households with replacement), and the variance of these 5000 estimates provided the uncertainty of the unweighted estimates. Finally, the variability associated with the multiple imputation procedure was added to the variance of the (weighted and unweighted) sero-prevalence estimates following the approach detailed in Honaker et al. (2011) [30]. In short, the final variance estimate $V$ is a combination of the average of the variance estimates ${V}_{j}, j = 1,\dots , m$ (described above) over the *m* replications and the variance of the *m* sero-prevalence estimates ${\theta }_{j}, j = 1, \dots , m$:

$$V=\frac1m{\textstyle\sum_{j=1}^m}V_j+S^2\left(1+\frac1m\right),with\,S^2=\frac1{m-1}{\textstyle\sum_{j=1}^m}\left(\theta_j-\overline\theta\right)^2$$

The final sero-prevalence estimates were obtained using the means of the *m* estimates, and 95% confidence intervals were computed assuming a normal distribution.

Breakthrough infections (BTI) are defined as newly infected participants after vaccination. The corresponding SARS-CoV-2-related serological spectrum is hence given by: anti-N negative but anti-S positive in the past and anti-N positive for a given next round (Fig. 1B). Accordingly, newly anti-N positive cases without anti-S antibodies in the previous rounds were defined as infections of naïve subjects (INS). While these estimates could be adjusted for the sensitivity and specificity of the test, we report in the results Sect. 95% confidence intervals (CI) for the ratio INS/BTI without adjustment. Indeed, the calculation of the variance requires information at the individual level (enabling accounting for the sampling design, the non-response, the calibration and the multiple imputation), while the adjustment of the incidence rates is done directly on the estimates.

Of interest were also risk factors for infection, with the aim to model when, in the course of the pandemic period, the infection (anti-N positiveness) occurred. Right censoring was adopted for anti-N negative participants at the end of the observation period, Follow-up 4. An extended Cox regression model [31, 32] was applied to assess which baseline risk factors increase or decrease the risk of infection. Since positivity of individuals in one household might depend on each other (resulting in a potential high intra-cluster correlation [33]), the Cox regression model follows the count process formulation of Anderson and Gill [31] to adjust for intra-household clustering in the data obtaining robust standard error estimates.

The non-response mechanism (Fig. 2B) over the different rounds of interrogation was studied using a logistic regression. The missingness in the explanatory variables was corrected by multiple imputation with *m* = 5 replications (Table 2). Due to a high number of missing values on the income (Supplemental Figure S1), a sensitivity analysis was performed considering complete cases for all covariates, except for the income where an indicator variable for missingness was used (Supplemental Table S1). The results are similar between the two analyses.

**Table 2:** Non-response mechanism at the different follow-ups using multiple imputation

Variable	Categories	Follow-up 2			Follow-up 3			Follow-up 4		
		OR	95% CI	*p*-value	OR	95% CI	*p*-value	OR	95% CI	*p*-value
Sex	Male	0.81	[0.66; 0.98]	*	0.97	[0.82; 1.15]		0.83	[0.69; 0.99]	*
Age *(years)*	14–19	0.82	[0.49; 1.37]		0.59	[0.36; 0.97]	*	0.61	[0.36; 1.05]	
	20–34	0.59	[0.45; 0.76]	***	0.55	[0.43; 0.69]	***	0.62	[0.49; 0.78]	***
	35–49	0.86	[0.67; 1.11]		1.02	[0.81; 1.30]		0.89	[0.70; 1.15]	
	50–64	1.47	[1.15; 1.88]	**	1.41	[1.13; 1.76]	**	1.57	[1.24; 1.98]	***
	65–79	1.87	[1.36; 2.58]	***	2.01	[1.46; 2.75]	***	1.28	[0.95; 1.71]	
	80 +	0.88	[0.57; 1.35]		1.06	[0.69; 1.63]		1.48	[0.96; 2.28]	
Birth country	Not Germany	0.98	[0.76; 1.27]		0.59	[0.47; 0.74]	***	0.63	[0.50; 0.79]	***
Level of education	In school	1.00	[0.58; 1.73]		0.88	[0.52; 1.50]		1.00	[0.57; 1.76]	
	$<$ 12 years	0.94	[0.69; 1.27]		1.10	[0.83; 1.46]		0.93	[0.69; 1.24]	
	$\ge$ 12 years	1.06	[0.78; 1.45]		1.03	[0.77; 1.37]		1.08	[0.78; 1.48]	
Employment status	Employed	1.07	[0.86; 1.32]		1.06	[0.87; 1.30]		0.98	[0.78; 1.22]	
	Self employed	0.89	[0.65; 1.23]		0.85	[0.65; 1.11]		0.90	[0.68; 1.20]	
	Unemployed	0.75	[0.57; 0.99]	*	1.27	[1.00; 1.62]		1.18	[0.90; 1.54]	
	Others	1.40	[0.84; 2.32]		0.87	[0.58; 1.31]		0.96	[0.59; 1.58]	
Risk employment	Yes	0.85	[0.63; 1.14]		1.10	[0.86; 1.41]		1.05	[0.82; 1.34]	
Smoking status	Non smoker	1.00	[0.87; 1.16]		1.17	[1.03; 1.33]	*	0.96	[0.84; 1.09]	
	Past smoker	0.92	[0.78; 1.09]		1.01	[0.87; 1.19]		1.00	[0.87; 1.15]	
	Current smoker	1.08	[0.91; 1.29]		0.84	[0.72; 0.98]	*	1.05	[0.89; 1.23]	
General health	Not good	0.61	[0.42; 0.88]	**	0.84	[0.60; 1.18]		0.59	[0.41; 0.85]	**
	Good	0.92	[0.73; 1.15]		1.08	[0.91; 1.28]		0.90	[0.75; 1.08]	
	Very good	1.28	[1.05; 1.54]	*	1.03	[0.88; 1.21]		1.19	[0.99; 1.44]	
	Excellent	1.39	[1.11; 1.76]	**	1.07	[0.86; 1.33]		1.57	[1.25; 1.97]	***
Respiratory allergies	Yes	0.92	[0.71; 1.19]		1.39	[1.10; 1.74]	**	0.83	[0.67; 1.02]	
Diabetes	Yes	1.37	[0.83; 2.28]		0.78	[0.48; 1.29]		0.81	[0.51; 1.30]	
CVD	Yes	1.10	[0.75; 1.60]		1.16	[0.87; 1.54]		1.15	[0.83; 1.58]	
Obesity	Yes	0.86	[0.50; 1.50]		0.89	[0.60; 1.31]		1.01	[0.67; 1.51]	
Cancer	Yes	0.87	[0.53; 1.43]		0.98	[0.58; 1.67]		1.02	[0.64; 1.63]	
Lung disease	Yes	0.93	[0.59; 1.45]		0.81	[0.58; 1.14]		0.97	[0.69; 1.36]	
Skin allergies	Yes	1.12	[0.81; 1.55]		0.98	[0.76; 1.27]		1.18	[0.90; 1.54]	
Autoimmune disease	Yes	1.28	[0.74; 2.22]		0.97	[0.68; 1.40]		1.34	[0.86; 2.08]	
Household type	Single	1.23	[0.93; 1.62]		1.25	[0.96; 1.62]		0.94	[0.73; 1.21]	
	Couple	1.24	[1.03; 1.49]	*	1.10	[0.94; 1.29]		1.19	[1.01; 1.40]	*
	Family	0.85	[0.69; 1.06]		0.86	[0.70; 1.06]		0.89	[0.73; 1.10]	
	Others	0.77	[0.61; 0.98]	*	0.85	[0.68; 1.06]		1.00	[0.78; 1.29]	
Household income *(Euro)*	≤ 2500	0.84	[0.67; 1.05]		0.81	[0.63; 1.04]		0.94	[0.75; 1.17]	
	2501–4000	1.01	[0.78; 1.30]		0.91	[0.76; 1.10]		0.92	[0.78; 1.08]	
	4001–6000	1.13	[0.95; 1.33]		1.16	[0.92; 1.46]		1.09	[0.92; 1.28]	
	6001 +	1.05	[0.76; 1.44]		1.16	[0.94; 1.44]		1.07	[0.87; 1.32]	
Living area/inhabitant *(sqm/individual)*	≤ 30	1.13	[0.92; 1.38]		0.97	[0.81; 1.17]		0.96	[0.80; 1.16]	
	31–40	1.03	[0.86; 1.23]		0.88	[0.75; 1.03]		1.02	[0.86; 1.21]	
	41–55	0.91	[0.74; 1.10]		1.27	[1.06; 1.51]	*	1.10	[0.91; 1.33]	
	56 +	0.95	[0.74; 1.22]		0.92	[0.74; 1.15]		0.92	[0.75; 1.15]	
Building type *(nb of apartments)*	1–2	1.24	[1.02; 1.51]	*	0.90	[0.76; 1.08]		1.11	[0.92; 1.33]	
	3–4	0.91	[0.70; 1.19]		1.48	[1.14; 1.91]	**	1.03	[0.80; 1.31]	
	5 +	0.88	[0.75; 1.04]		0.75	[0.64; 0.88]	***	0.88	[0.75; 1.03]	
Seropositivity in the previous rounds	Negative	4.52	[3.78; 5.40]	***	5.42	[4.74; 6.19]	***	5.27	[4.63; 6.01]	***
	Positive	2.01	[1.48; 2.72]	***	1.88	[1.54; 2.30]	***	1.90	[1.57; 2.31]	***
	Missing	0.11	[0.09; 0.13]	***	0.10	[0.08; 0.12]	***	0.10	[0.09; 0.12]	***

Variables with 2 categories have contrasts with one category set to 0. For variables with 3 and more categories, constraint sum-to-zero contrasts was applied

*OR* odds ratio

p-value: *** p < 0.001; ** p < 0.01; * p < *0.05*

In both the risk factor analysis and the non-response mechanism analysis, for explanatory variables with two categories, a constraint to zero for one category (e.g. females vs. males) was used. For covariates with three and more categories, a sum-to-zero constraint (i.e. compare each category to the average) was applied.

## Results

### Cohort development

Since anti-S becomes positive after vaccination but also after infection, the definition of being vaccinated for infected persons was obtained using the questionnaires when available (Fig. 1B). When describing the changes of antibody statuses over time, historical information needs to be taken into account. Figure 2A applies the definition of „ever positiveness “ (see Supplemental Figure S2 for an alternative serological description) and considers the following major categories: only infected (anti-N ever positive, and vaccination excluded based on other information), naïve (anti-N and anti-S never positive), vaccinated (only anti-S ever positive), and infected & vaccinated (anti-N positive after anti-S positive, or anti-N positive with respective questionnaire information). From Follow-up 2 on, participants started moving from the naïve to the vaccinated status, which became the most prominent stage in Follow-ups 3 and 4. The status of non-responders is labelled as missing: 64% (3396/5313) of the participants gave blood in all rounds, 11% (578/5313) / 8% (401/5313) / 6% (332/5313) had exactly one/two/three rounds missing, and 11% (606/5313) dropped out for all four follow-ups after the baseline measurement (Fig. 2B). Some non-responders still answered back in subsequent round(s), thus moving away from stage missing. Overall, the response rate was satisfactory (83% Follow-up 1; 82% Follow-up 2; 73% Follow-up 3; 71% Follow-up 4; Fig. 2B), especially considering the duration of the cohort.

### Non-responder analyses

The non-response mechanism for the Follow-up 1 was previously presented [20]. We show the results for the last three follow-ups (Table 2). Females and participants between 50 and 79 years were more likely to take part to the follow-ups, while young participants (age < 35 years old) together with participants with a migration background were less likely to participate. People who reported a bad general health condition tended to drop out of the cohort while those with excellent health continued answering to the survey. Couples were slightly more likely to provide blood samples than other household types. Members of a household with a low or medium-to-low income were less likely to take part in the survey in comparison to households with a medium-to-high or high income, even though the differences were not significant (see Supplemental Table S1 for sensitivity analysis). During Follow-up 2, households in buildings with 1–2 apartments tended to answer more often, while during Follow-up 3, those living in buildings with 3–4 apartments answered more often. Households in buildings with 5 or more apartments answered less often. Participants not taking part in one previous round of interrogation were less likely to take part in the next rounds. Having at least one positive anti-N serological result in the previous rounds lead to a lower response rate in the next follow-ups in comparison to always having negative anti-N results in the past. All other covariates investigated in the non-response mechanism (level of education, employment status, smoking status, etc.) showed no or negligible association to the response behaviour.

### SARS-CoV-2 sero-prevalence, underreporting factor and sero-incidence over time

**Fig. 3:**
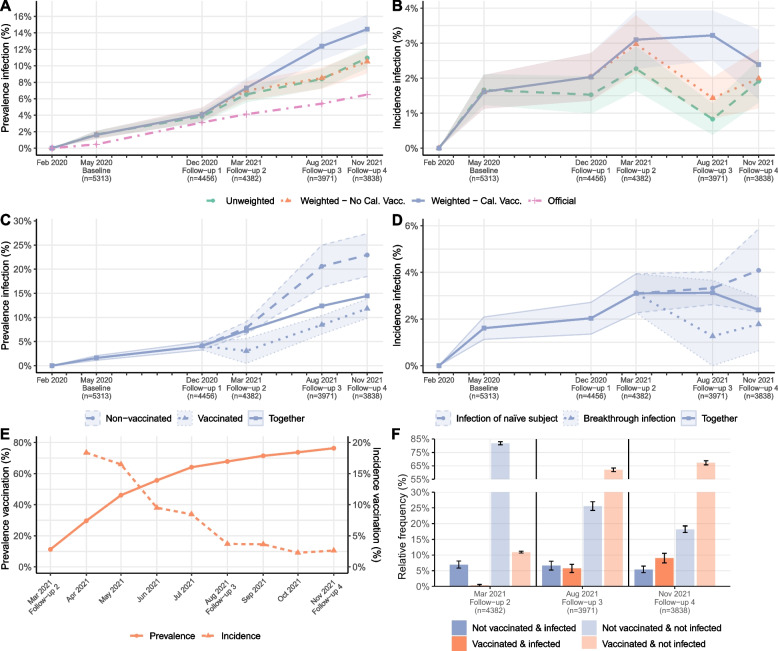
**A** Weighted and unweighted cumulative anti-N sero-prevalence in private households and official numbers of cases reported by the authorities for the Munich population older than 13 years. **B** Weighted and unweighted anti-N sero-incidence. **C** Anti-N sero-prevalence estimates calibrated on the number of vaccinated people split according to the vaccination status of the same round. **D** Calibrated estimates for the infection of naïve subjects and breakthrough infections. **E** Prevalence and incidence of vaccination in Munich (official numbers). **F** Relative frequencies according to the infection and vaccination status

The blue estimate in Fig. 3A shows the calibrated cumulative sero-prevalence (adjusted for sensitivity and specificity) in private households for the Munich population 14 years and older:

- Baseline: 1.6% (1.1 – 2.1%),
- Follow-up 1: 4.1% (3.3%—4.9%), and after adjustment for vaccination status
- Follow-up 2: 7.3% (6.1—8.5%),
- Follow-up 3: 12.4% (10.7—14.1%),
- Follow-up 4: 14.5% (12.7—16.2%).

Without adjustment for vaccination status for the Follow-ups 3 and 4, the sero-prevalence would have been significantly lower: 8.5% (7.2-9.8%) for August 2021 and 10.5% (9.1-11.9%) for November 2021. Indeed, the proportion of vaccinated persons is greater in the cohort in comparison to the general Munich population. Therefore, the calibration on the vaccination status increases the weight of the participants who are not vaccinated. The sero-prevalence being greater in the non-vaccinated population (see below and Fig. 3C), the overall sero-prevalence, including both vaccinated and non-vaccinated, also increases with the calibration.

The official number of positive cases is reported in pink in Fig. 3A for the general population of Munich (including institutions like nursing homes and potential reinfections). Considering that the KoCo19 cohort is limited to private households and that the estimated sero-prevalence does not account for multiple infections, a comparison of this estimate with the official number over time allows us to estimate a lower bound for the underreporting factor (with the false assumption that all cases reported by the authorities occurred in private households and neglecting reinfections). The estimated underreporting factor changed over the rounds:

- Baseline: 3.4 (2.4 – 4.4),
- Follow-up 1: 1.3 (1.0 – 1.6),
- Follow-up 2: 1.8 (1.5 – 2.1),
- Follow-up 3: 2.3 (2.0 – 2.6),
- Follow-up 4: 2.2 (2.0—2.5).

Figure 3B depicts the sero-incidence (adjusted for sensitivity and specificity), i.e. the percentage of new infections between two consecutive rounds:

- Follow-up 1: 2.0% (1.4—2.7%),
- Follow-up 2: 3.1% (2.3—3.9%),
- Follow-up 3: 3.2% (2.5—3.9%),
- Follow-up 4: 2.4% (1.4—3.4%),

with the time interval between Follow-ups 3 and 4 being rather short (three months).

### Breakthrough infections in the Munich population

To better understand the effect of the vaccination campaign (see also next section), the calibrated cumulative sero-prevalence was split between vaccinated versus non-vaccinated people (Fig. 3C):

- Follow-up 2: 3.1% (0.5% - 5.6%) versus 7.8% (6.6 – 9.1%),
- Follow-up 3: 8.5% (6.6 – 10.4%) versus 20.6% (16.2 - 25.0%) and
- Follow-up 4: 11.8% (9.8 - 13.8%) versus 22.9% (18.5 - 27.4%).

The sero-prevalence of the vaccinated group is lower compared to the non-vaccinated group.

Figure 3D compares the adjusted (for sensitivity and specificity) incidence rates for BTI versus INS over the rounds:

- Follow-up 3: 1.3% (0 - 3.7%) versus 3.3% (2.6 - 4%) and
- Follow-up 4: 1.8% (0.6 - 2.9%) versus 4.1% (2.3 - 5.9%).

In August and November 2021, incidence rates of INS were greater than the ones of BTI. Significant differences between unadjusted INS and BTI incidence rates (INS/BTI) could however not be achieved:

- Follow-up 3: ratio of 2.8 (0 - 7.7) and
- Follow-up 4: 2.1 (0.7 - 3.6).

The low sample sizes led to low power and may thus have implied the non-significant findings: In Follow-up 2, the low number of vaccinated persons led to high uncertainty in the estimation of BTI in Follow-up 3; vice versa, in Follow-up 3, the low number of non-vaccinated persons led to high uncertainty in the estimation of INS in Follow-up 4.

### The vaccination campaign in the Munich population

The introduction of vaccination quickly changed the SARS-CoV-2-related serological spectrum of the Munich population. The percentage of the Munich population presenting antibodies against the virus (either anti-S after infection and/or vaccination and/or anti-N antibodies after infection) increased fast over time:

- Follow-up 2: 11.2% (9.6 - 12.8%),
- Follow-up 3: 74.2% (72.6 – 75.8%),
- Follow-up 4: 86.8% (85.8 - 87.9%).

Even though the cumulative sero-prevalence and the sero-incidence seemed to be higher among the non-vaccinated population compared to the vaccinated population (Fig. 3C and D), BTI contributed relevantly to the community spread, considering that the size of the population of vaccinated people was much larger than the non-vaccinated one during the last rounds of interrogation (Fig. 3E). Figure 3F illustrates this effect in more detail. The proportion of people vaccinated and infected increased over time, up to Follow-up 4 where this proportion was significantly greater than the one of infected and non-vaccinated people. This figure also shows that the proportion of the population without any antibodies related to SARS-CoV-2 (non-vaccinated and non-infected) was decreasing over time, while the share of people vaccinated and non-infected increased (cf. Fig. 2A).

### Risk factors for SARS-CoV-2 sero-prevalence

**Fig. 4:**
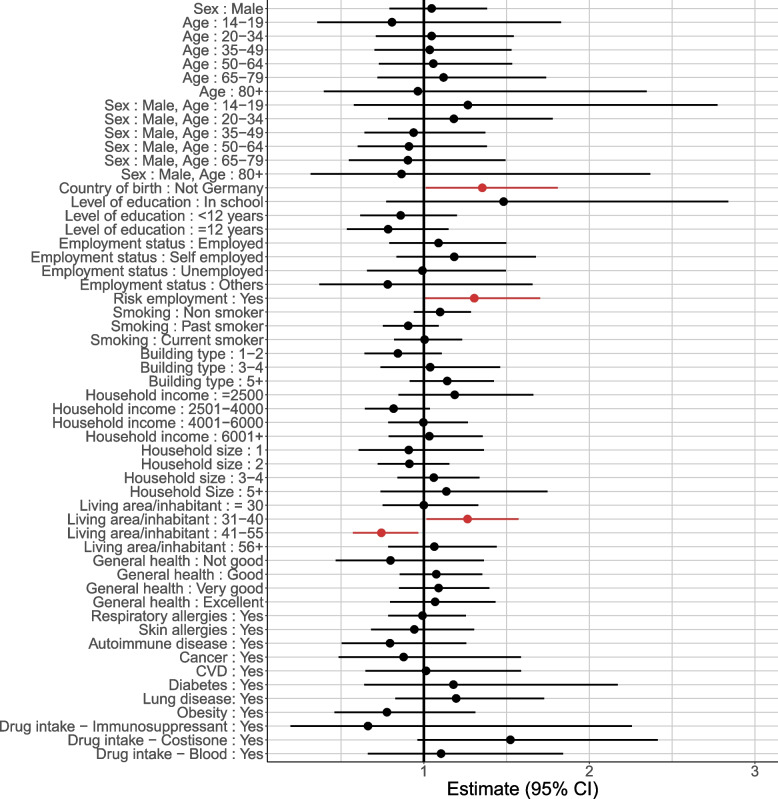
Association between potential risk factors and SARS-CoV-2 sero-positivity taking into account time between baseline and Follow-up 4; events are thus right-censored. Results are based on multiple imputation. The main individual level risk factors were country of birth outside Germany and being employed in a job more in contact with the epidemic. Living in an apartment with a living area of 30–40 square meters per inhabitant revealed a slightly higher risk, while for 40–55 square meters per inhabitant the hazard ratio decreased

The results of the risk factor analysis can be found in Fig. 4. The extended Cox regression model suggests that being born outside Germany (hazard ratio (HR) 1.36, 95% confidence interval (CI) 1.01–1.85) and having a job with a high potential of contact to COVID-19 cases (HR 1.31, 95% CI 1.00–1.70) were risk factors for SARS-CoV-2 sero-positivity. Living area of 30–40 square meters per inhabitant presented a slightly higher risk of infection (HR 1.27, 95% CI 1.01–1.59), while for 40–55 square meters per inhabitant the risk decreased (HR 0.74, 95% CI 0.57–0.97), compared to the average Hazard of all categories of living area. All other socio-demographic (sex, age, level of education, employment status, building type, household income) and health-related variables (smoking status, general health status, different diseases and drug intakes) were not identified as risk factors for infection.

### Household and neighbourhood clustering of SARS-CoV-2 cases

SARS-CoV-2 transmission within households was found to be highly significant for baseline [33] and Follow-up 1 [20] analyses and was confirmed until Follow-up 4 (Supplemental Figure S3). While the overall picture obtained in recent rounds showed a lower-than-expected mean variance at 500 m as well, we now could not find sufficient proof of spatial clustering beyond household level, especially if one adjusted *p*-values for multiple testing.

## Discussion

We present the development of the SARS-CoV-2 pandemic in the municipality of Munich. To estimate the real number of SARS-CoV-2 infections, the members of the prospective KoCo19 cohort were asked five times to give their blood for study purposes between spring 2020 and fall 2021. SARS-CoV-2 antibodies generated by silent or symptomatic infections and/or vaccination could hence be measured. We could show that the sero-prevalence drastically increased over time, from 1.6% during the baseline to 14.5% in Follow-up 4, with a relevant underreporting bias. Risk factors for SARS-CoV-2 sero-positivity, such as being born outside of Germany, living area per inhabitant and working in a job with high potential of contact with COVID-19, could be identified together with household clustering.

Sero-prevalence was still low towards the end of the first pandemic wave and increased drastically in every follow-up. Comparison of our results with official numbers reveals an underreporting factor that changes over time. These changes might result from different testing policies as well as different variants of the virus. The estimates present lower bounds of the true underreporting factor, since our study focused on private households whereas the official number of reported cases included institutions (like nursing homes) as well. Moreover, potential reinfections counted in the official numbers were here neglected. Indeed, our study focuses on the pandemic from its beginning to the Delta variant, before the spread of the Omicron variant. Therefore, the low number of reinfections did not play a major role during this period [34–36].

In our data it was possible to separate infection of naïve subjects from breakthrough infections in low- and high-incidence time periods. In all follow-ups, our results indicate a contribution of breakthrough infections to the spread of SARS-CoV-2. The findings presented here, based on serology, contribute to current knowledge so far derived from PCR test results. The number of breakthrough infections detected based on PCR tests that were either done routinely, because of symptoms or among case contacts [37, 38] might miss an important number of silent infections, especially as vaccinated individuals tend to have less pronounced symptoms. In our cohort, only a small part was fully vaccinated until March 2021 (Follow-up 2), given the vaccination scheme in Germany at that time. This resulted in a wide confidence interval for breakthrough infections during the next follow-up. During August 2021 (Follow-up 3), almost the complete cohort got vaccinated and therefore, the estimation uncertainty for breakthrough infections during Follow-up 4 decreased. 99.4% of the people stating vaccination in the questionnaire sero-converted in anti-S, indicating a good efficacy of the vaccinations. In concordance with other studies [39, 40], a considerable proportion of breakthrough infections was detected. Our results as well as other studies suggest that vaccination lowers the risk of infection [41]. Moreover, the share of infected persons (sero-prevalence) was shown to be greater in the non-vaccinated population in comparison to the vaccinated one. The sero-incidence of (most likely asymptomatic) infections among vaccinated people in the population was lower than the one in non-vaccinated people; however, the difference was statistically non-significant. BTIs might thus relevantly contribute to the community spread, considering also the fact that the vaccinated population was much larger compared to the non-vaccinated one. This might be even more relevant for highly transmissible variants like Omicron.

With an increasing prevalence of vaccination in the population, silent infections or persons presenting only mild symptoms are common. In this context, population-based sero-prevalence studies are important to estimate the true population prevalence. A couple of German cross-sectional population-based sero-prevalence studies were published especially during the first and second wave of the pandemic [42–44]. To our knowledge, all these studies stopped by mid 2021, leaving our cohort as the only one.

In our first analysis [33], an increased (albeit not statistically significant) risk of infection of having a job with a high potential of contact to COVID-19 cases could be found. With this analysis the risk factor became statistically significant, which is in line with other studies [45–47]. The World Health Organisation reported that among the COVID-19 cases reported worldwide, 14% belong to the group of healthcare workers, whereas in most countries this group represents less than 3% of the general population [48].

Participants with a living area between 31 and 40 square meters per inhabitant showed a significantly increased risk for infection, while the risk of the group with a living area between 41 and 55 square meters per inhabitant significantly decreased. Considering the number of household members, we found that 56% (76%) of the households with 31 - 40 (41—55) squared meters per inhabitant also have only one or two household members. Knowing that a larger household size implies more possible infectious contacts [49–51] suggests that the risk also depends on the household composition: Less members are associated to lower risk of infection. Household size is included in the model but does not show any significant effect, also not as interaction term, although the risk of infection seems to become higher with more household members (Fig. 4). This might be due to the fact that the variables household size, living area per inhabitant and building type all describe the living situation, with difficulties in separating the risk effects. Nevertheless, no multicollinearity issues were detected for this analysis.

Beside the two aforementioned risks for infection and being born outside Germany, no other socio-demographic or health-related risk factors were identified in our study. These results should rather be seen as exploratory than confirmatory, considering that we made no adjustment for multiple testing.

Major strengths of our study are its population-based approach, the appropriate weighting of results for the general Munich population, the high number of participants, the thorough validation of the assays used, and the use of validated questionnaire items. The overall response to the study was high compared to other population-based epidemiological studies in Germany (64% of the participants gave specimens in all rounds) [52]. While most participants completed the questionnaire online or on paper, we also provided the alternative of telephone interviews, which helped increasing participation. A relevant limitation of our study is the exclusion of children and residents not living in private households. While in general, people with migration background are less likely to participate in population-based studies, the lack of translated questionnaires further limited the number of migrants participating in our study [21]. To increase response, blood samples were collected at participants’ homes or via mail with the DBS introduction and not at a centralized testing facility. Although until now a lot of research has been done for the COVID-19 pandemic, definitions like correlate of protection and long COVID symptoms are still not fully understood. Therefore, we aim to continue our longitudinal prospective representative cohort.

## Conclusion

Despite the vaccination campaign, SARS-CoV-2 sero-prevalence in the Munich general population increased drastically towards the end of 2021, but was still below 20%. The estimated number of infected persons was nevertheless at least twice as high as the official number reported by the authorities during the second half of 2021. Workers with a high potential of contact to infected persons experienced an increased risk of infection. Breakthrough infections still contribute to the community spread, thus we conclude that non-pharmaceutical interventions are still relevant, especially in the presence of highly transmissible variants like Omicron.

## Supplementary Information

**Additional file 1:** **Figure S1.** Missing pattern in the baseline questionnaire. Bottom middle: variable analysed for missing information. Bottom left: bar chart depicting numbers of missing information for that variable. Bottom right: description of intersection pattern between variables (all possible combinations of the variables for which a missing information was given, from left to right e.g. only income information missing, income & living & household type information missing, all variables missing, etc.). Top: bar chart depicting the numbers of participants that did not give information for that intersection pattern.

**Additional file 2:** **Figure S2.** Cohort description based on current lab result (in contrast to ever-positivity as in Figure 2). Change of serological status of participants: only infected (anti-N positive and stated to be non-vaccinated in the questionnaire), naïve (anti-N and anti-S negative), vaccinated (only anti-S positive), infected & vaccinated (anti-N positive and in previous round only anti-S positive, or anti-N positive and stated to be vaccinated in the questionnaire), infected without information on vaccination status (infected, undefined vaccination) and non-responders/missing.

**Additional file 3:** **Figure S3.** Proximity cluster analysis at Follow-ups 2 to 4. The grey points and curves show the distribution of mean within-cluster variances for 10,000 random permutations of cluster assignments. The horizontal lines show the observed values. Cluster variables are households, buildings, and geospatial clusters of different sizes. Household membership was left invariant when considering buildings and geospatial clusters. p-values indicate the one-sided probability of observing smaller than observed values under random cluster assignments. Results indicate within-household clustering and suggest neighbourhood transmission only in the cluster with 500m.

**Additional file 4:** **Table S1.** Non-response mechanism at the different follow-ups using complete cases and indicator of missingness for income.

## Data Availability

Our data are accessible to researchers upon reasonable request to the corresponding author taking data protection laws and privacy of study participants into account. To facilitate reproducibility and reuse, the analysis and figure generation code has been made available on GitHub (https://github.com/koco19/epi3) and will be uploaded to ZENODO for long-term storage.

## Abbreviations

anti-N: Anti-Nucleocapsid antibodies
anti-S: Anti-Spike antibodies
BTI: Breakthrough infections
CI: Confidence interval
DBS: Dry blood spot
HR: Hazard ratio
INS: Infections of naïve subjects
KoCo19: Representative COVID-19 Cohort Munich
PCR: Polymerase chain reaction
Ro-N-Ig: Elecsys® Anti-SARS-CoV-2 anti-N (Roche)
Ro-RBD-Ig: Elecsys® Anti-SARS-CoV-2 anti-S (Roche)
RT-PCR: Reverse transcription polymerase chain reaction
SARS-CoV-2: SARS corona virus 2
