# Supplementary material for: The representative COVID-19 cohort Munich (KoCo19): from the beginning of the pandemic to the Delta virus variant
Source: BMC Infect Dis. 2023 Jul 13;23:466. doi: 10.1186/s12879-023-08435-1 (PMC10339498; doi:10.1186/s12879-023-08435-1)

**Figure S1**. Missing pattern in the baseline questionnaire. Bottom middle: variable analysed for missing information. Bottom left: bar chart depicting numbers of missing information for that variable. Bottom right: description of intersection pattern between variables (all possible combinations of the variables for which a missing information was given, from left to right e.g. only income information missing, income & living & household type information missing, all variables missing, etc.). Top: bar chart depicting the numbers of participants that did not give information for that intersection pattern.


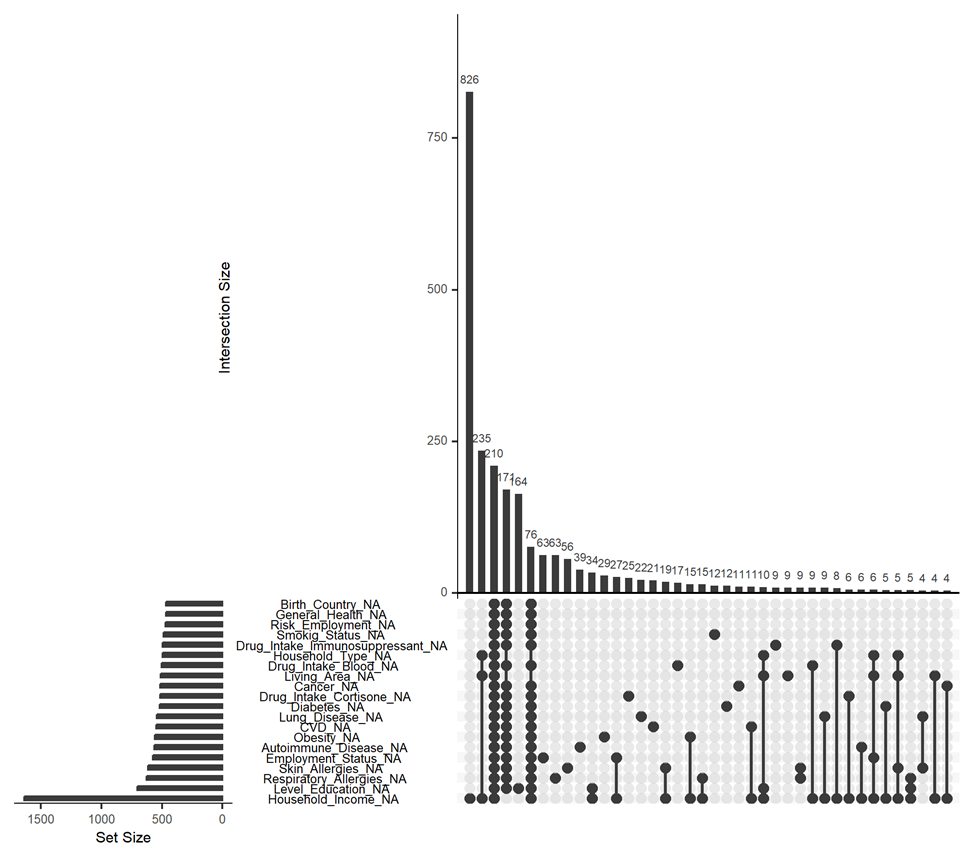

Supplement: Supplementary file 1 — Additional file 1: Figure S1. Missing pattern in the baseline questionnaire. Bottom middle: variable analysed for missing information. Bottom left: bar chart depicting numbers of missing information for that variable. Bottom right: description of intersection pattern between variables (all possible combinations of the variables for which a missing information was given, from left to right e.g. only income information missing, income & living & household type information missing, all variables missing, etc.). Top: bar chart depicting the numbers of participants that did not give information for that intersection pattern. [file 12879_2023_8435_MOESM1_ESM.docx]
