# Supplementary material for: The representative COVID-19 cohort Munich (KoCo19): from the beginning of the pandemic to the Delta virus variant
Source: BMC Infect Dis. 2023 Jul 13;23:466. doi: 10.1186/s12879-023-08435-1 (PMC10339498; doi:10.1186/s12879-023-08435-1)
Supplement: Supplementary file 2 — Additional file 2: Figure S2. Cohort description based on current lab result (in contrast to ever-positivity as in Figure 2). Change of serological status of participants: only infected (anti-N positive and stated to be non-vaccinated in the questionnaire), naïve (anti-N and anti-S negative), vaccinated (only anti-S positive), infected & vaccinated (anti-N positive and in previous round only anti-S positive, or anti-N positive and stated to be vaccinated in the questionnaire), infected without information on vaccination status (infected, undefined vaccination) and non-responders/missing. [file 12879_2023_8435_MOESM2_ESM.docx]

**Figure S2**. Cohort description based on current lab result (in contrast to ever-positivity as in Figure 2). Change of serological status of participants: only infected (anti-N positive and stated to be non-vaccinated in the questionnaire), naïve (anti-N and anti-S negative), vaccinated (only anti-S positive), infected & vaccinated (anti-N positive and in previous round only anti-S positive, or anti-N positive and stated to be vaccinated in the questionnaire), infected without information on vaccination status (infected, undefined vaccination) and non-responders/missing.
