# Supplementary material for: The representative COVID-19 cohort Munich (KoCo19): from the beginning of the pandemic to the Delta virus variant
Source: BMC Infect Dis. 2023 Jul 13;23:466. doi: 10.1186/s12879-023-08435-1 (PMC10339498; doi:10.1186/s12879-023-08435-1)

**Figure S3**: Proximity cluster analysis at Follow-ups 2 to 4. The grey points and curves show the distribution of mean within-cluster variances for 10,000 random permutations of cluster assignments. The horizontal lines show the observed values. Cluster variables are households, buildings, and geospatial clusters of different sizes. Household membership was left invariant when considering buildings and geospatial clusters. p-values indicate the one-sided probability of observing smaller than observed values under random cluster assignments. Results indicate within-household clustering and suggest neighbourhood transmission only in the cluster with 500m.


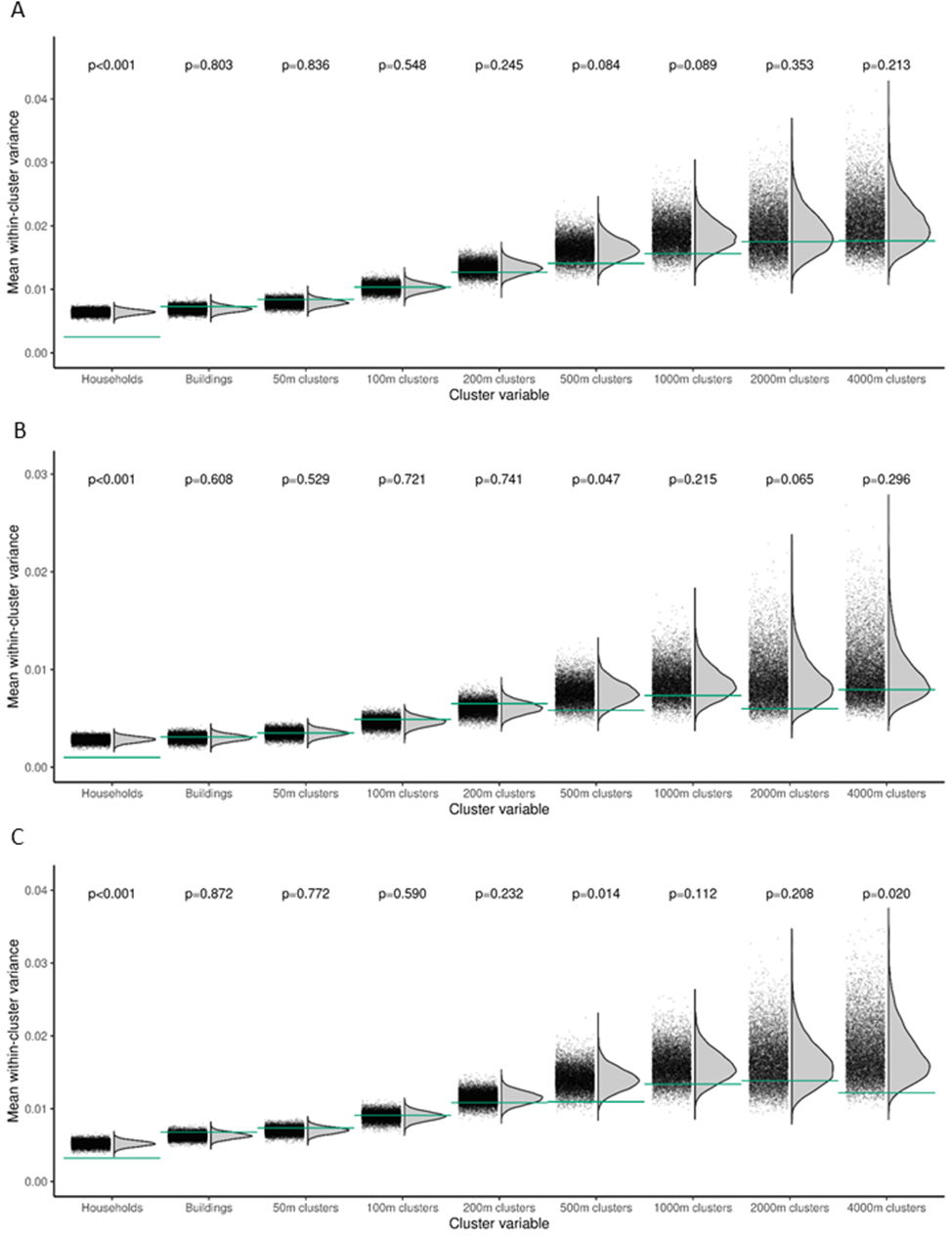

Supplement: Supplementary file 3 — Additional file 3: Figure S3. Proximity cluster analysis at Follow-ups 2 to 4. The grey points and curves show the distribution of mean within-cluster variances for 10,000 random permutations of cluster assignments. The horizontal lines show the observed values. Cluster variables are households, buildings, and geospatial clusters of different sizes. Household membership was left invariant when considering buildings and geospatial clusters. p-values indicate the one-sided probability of observing smaller than observed values under random cluster assignments. Results indicate within-household clustering and suggest neighbourhood transmission only in the cluster with 500m. [file 12879_2023_8435_MOESM3_ESM.docx]
