# Supplementary material for: The representative COVID-19 cohort Munich (KoCo19): from the beginning of the pandemic to the Delta virus variant
Source: BMC Infect Dis. 2023 Jul 13;23:466. doi: 10.1186/s12879-023-08435-1 (PMC10339498; doi:10.1186/s12879-023-08435-1)
Supplement: Supplementary file 4 — Additional file 4: Table S1. Non-response mechanism at the different follow-ups using complete cases and indicator of missingness for income. [file 12879_2023_8435_MOESM4_ESM.zip › 1-Table S1.docx]

**Table S1**. Non-response mechanism at the different follow-ups using complete cases and indicator of missingness for income.

| **Variable** | **Categories** | **Follow-up 2** | | | **Follow-up 3** | | | **Follow-up 4** | | |
| --- | --- | --- | --- | --- | --- | --- | --- | --- | --- | --- |
|  |  | OR | 95% CI | p-value | OR | 95% CI | p-value | OR | 95% CI | p-value |
| Sex | Male | 0.76 | [0.59; 0.97] | * | 0.99 | [0.80; 1.22] |  | 0.85 | [0.68; 1.05] |  |
| Age  *(years)* | 14-19 | 0.86 | [0.46; 1.60] |  | 0.61 | [0.35; 1.08] |  | 0.75 | [0.39; 1.43] |  |
|  | 20-34 | 0.49 | [0.36; 0.67] | *** | 0.69 | [0.53; 0.92] | ** | 0.61 | [0.46; 0.82] | ** |
|  | 35-49 | 0.77 | [0.55; 1.06] |  | 1.04 | [0.79; 1.37] |  | 0.85 | [0.64; 1.14] |  |
|  | 50-64 | 1.63 | [1.18; 2.25] | ** | 1.57 | [1.20; 2.05] | ** | 1.51 | [1.14; 2.00] | ** |
|  | 65-79 | 1.83 | [1.22; 2.74] | ** | 1.73 | [1.20; 2.48] | ** | 1.15 | [0.80; 1.65] |  |
|  | 80+ | 1.05 | [0.61; 1.79] |  | 0.84 | [0.52; 1.35] |  | 1.47 | [0.88; 2.47] |  |
| Birth country | Not Germany | 0.99 | [0.72; 1.37] |  | 0.67 | [0.51; 0.87] | ** | 0.62 | [0.47; 0.81] | *** |
| Level of education | In school | 1.02 | [0.53; 1.97] |  | 0.89 | [0.50; 1.58] |  | 0.88 | [0.46; 1.67] |  |
|  | $<$ 12 years | 0.89 | [0.62; 1.27] |  | 1.15 | [0.84; 1.57] |  | 1.03 | [0.73; 1.45] |  |
|  | $\geq$ 12 years | 1.10 | [0.77; 1.59] |  | 0.98 | [0.71; 1.35] |  | 1.11 | [0.78; 1.57] |  |
| Employment status | Employed | 1.09 | [0.83; 1.43] |  | 1.05 | [0.84; 1.31] |  | 0.99 | [0.78; 1.26] |  |
|  | Self employed | 0.96 | [0.65; 1.41] |  | 0.85 | [0.62; 1.16] |  | 0.83 | [0.60; 1.15] |  |
|  | Unemployed | 0.65 | [0.47; 0.90] | ** | 1.22 | [0.92; 1.62] |  | 1.30 | [0.96; 1.74] |  |
|  | Others | 1.47 | [0.81; 2.68] |  | 0.92 | [0.56; 1.51] |  | 0.94 | [0.55; 1.59] |  |
| Risk employment | Yes | 1.00 | [0.71; 1.41] |  | 0.94 | [0.72; 1.24] |  | 1.08 | [0.81; 1.45] |  |
| Smoking status | Non smoker | 1.06 | [0.89; 1.26] |  | 1.09 | [0.95; 1.26] |  | 0.92 | [0.79; 1.07] |  |
|  | Past smoker | 0.83 | [0.69; 1.01] |  | 1.02 | [0.87; 1.20] |  | 1.08 | [0.91; 1.28] |  |
|  | Current smoker | 1.14 | [0.91; 1.41] |  | 0.90 | [0.75; 1.07] |  | 1.01 | [0.83; 1.22] |  |
| General health | Not good | 0.53 | [0.34; 0.83] | ** | 0.74 | [0.49; 1.13] |  | 0.51 | [0.34; 0.77] | ** |
|  | Good | 0.89 | [0.71; 1.12] |  | 1.13 | [0.92; 1.39] |  | 0.89 | [0.73; 1.09] |  |
|  | Very good | 1.38 | [1.09; 1.73] | ** | 1.08 | [0.88; 1.31] |  | 1.23 | [1.00; 1.51] | * |
|  | Excellent | 1.53 | [1.15; 2.04] | ** | 1.11 | [0.87; 1.41] |  | 1.79 | [1.38; 2.32] | *** |
| Respiratory allergies | Yes | 0.98 | [0.74; 1.30] |  | 1.37 | [1.07; 1.75] | * | 0.85 | [0.67; 1.10] |  |
| Diabetes | Yes | 1.42 | [0.76; 2.66] |  | 0.69 | [0.40; 1.19] |  | 0.73 | [0.42; 1.27] |  |
| CVD | Yes | 1.16 | [0.78; 1.72] |  | 1.12 | [0.80; 1.57] |  | 1.01 | [0.72; 1.42] |  |
| Obesity | Yes | 0.72 | [0.42; 1.24] |  | 0.95 | [0.59; 1.53] |  | 1.26 | [0.76; 2.10] |  |
| Cancer | Yes | 1.08 | [0.58; 2.00] |  | 1.02 | [0.61; 1.73] |  | 1.09 | [0.63; 1.87] |  |
| Lung disease | Yes | 1.08 | [0.68; 1.74] |  | 0.83 | [0.56; 1.22] |  | 0.87 | [0.59; 1.29] |  |
| Skin allergies | Yes | 1.21 | [0.83; 1.76] |  | 1.04 | [0.75; 1.43] |  | 1.27 | [0.91; 1.76] |  |
| Autoimmune disease | Yes | 1.88 | [1.03; 3.43] | * | 0.98 | [0.64; 1.49] |  | 1.53 | [0.96; 2.45] |  |
| Household type | Single | 1.23 | [0.87; 1.74] |  | 1.30 | [0.96; 1.76] |  | 1.16 | [0.85; 1.59] |  |
|  | Couple | 1.29 | [1.04; 1.61] | * | 1.07 | [0.89; 1.29] |  | 1.16 | [0.95; 1.40] |  |
|  | Family | 0.74 | [0.57; 0.95] | * | 0.91 | [0.73; 1.13] |  | 0.78 | [0.62; 0.98] | * |
|  | Others | 0.85 | [0.63; 1.15] |  | 0.79 | [0.61; 1.03] |  | 0.95 | [0.72; 1.26] |  |
| Household income  *(Euro)* | ≤ 2500 | 0.87 | [0.65; 1.18] |  | 0.75 | [0.58; 0.98] | * | 0.96 | [0.73; 1.28] |  |
|  | 2501-4000 | 0.96 | [0.74; 1.24] |  | 1.01 | [0.80; 1.26] |  | 0.80 | [0.64; 1.01] |  |
|  | 4001-6000 | 1.18 | [0.93; 1.50] |  | 1.26 | [1.03; 1.54] | * | 1.18 | [0.95; 1.45] |  |
|  | 6001+ | 1.30 | [1.00; 1.70] |  | 1.06 | [0.86; 1.31] |  | 1.23 | [0.98; 1.55] |  |
|  | Missing | 0.78 | [0.62; 0.97] | * | 0.98 | [0.81; 1.20] |  | 0.89 | [0.73; 1.09] |  |
| Living area/inhabitant  *(sqm/individual)* | ≤ 30 | 1.27 | [1.00; 1.62] |  | 0.95 | [0.78; 1.17] |  | 1.00 | [0.81; 1.23] |  |
|  | 31-40 | 0.92 | [0.75; 1.14] |  | 0.88 | [0.74; 1.06] |  | 0.96 | [0.80; 1.16] |  |
|  | 41-55 | 0.86 | [0.68; 1.09] |  | 1.27 | [1.02; 1.57] | * | 1.18 | [0.95; 1.47] |  |
|  | 56+ | 0.99 | [0.73; 1.35] |  | 0.94 | [0.72; 1.21] |  | 0.88 | [0.68; 1.15] |  |
| Building type  *(nb of apartments)* | 1-2 | 1.48 | [1.15; 1.89] | ** | 0.95 | [0.76; 1.19] |  | 1.06 | [0.85; 1.32] |  |
|  | 3-4 | 0.77 | [0.56; 1.06] |  | 1.53 | [1.10; 2.12] | * | 1.04 | [0.77; 1.42] |  |
|  | 5+ | 0.88 | [0.71; 1.08] |  | 0.69 | [0.57; 0.84] | *** | 0.91 | [0.75; 1.10] |  |
| Seropositivity in the previous rounds | Negative | 3.94 | [3.07; 5.06] | *** | 5.21 | [4.42; 6.14] | *** | 4.89 | [4.16; 5.75] | *** |
|  | Positive | 2.62 | [1.69; 4.06] | *** | 1.90 | [1.49; 2.43] | *** | 2.13 | [1.67; 2.72] | *** |
|  | Missing | 0.10 | [0.07; 0.13] | *** | 0.10 | [0.08; 0.12] | *** | 0.10 | [0.08; 0.12] | *** |
